# Targeting NSP16 Methyltransferase for the Broad-Spectrum Clinical Management of Coronaviruses: Managing the Next Pandemic

**DOI:** 10.3390/diseases9010012

**Published:** 2021-02-01

**Authors:** Ilham M. Alshiraihi, Gerald L. Klein, Mark A. Brown

**Affiliations:** 1 Cell and Molecular Biology Program, Colorado State University, Fort Collins, CO 80523, USA; alshiraihi@gmail.com; 2 Department of Biology, University of Tabuk, Tabuk 47713, Saudi Arabia; 3 MedSurgPI, Franklinton, NC 27525, USA; gklein@medsurgpi.com; 4 Department of Clinical Sciences, Colorado State University, Fort Collins, CO 80523, USA; 5 Epidemiology Section, Colorado School of Public Health, Fort Collins, CO 80523, USA; 6 Department of Ethnic Studies, Colorado State University, Fort Collins, CO 80523, USA; 7 Graduate Degree Program in Ecology, Colorado State University, Fort Collins, CO 80523, USA

**Keywords:** COVID-19, methyltransferase, coronavirus, mRNA capping

## Abstract

With the approval and distribution of demonstrably safe COVID-19 vaccines bearing exceptionally high efficacy profiles, it may be tempting to envision a return to “normal” in the coming months. However, if there is one lesson to be learned from the ongoing pandemic, it is that, in a world of evolving zoonotic viruses, we must be better prepared for the next deadly outbreak. While the acute nature of the COVID-19 pandemic demanded a highly specific approach, it is advisable to consider the breadth of seemingly endless possibilities in our approach to managing the next inevitable occurrence of an outbreak. Though there is little chance of discovering a “magic pill” to combat all future pathogens, the highly conserved nature of non-surface viral proteins exposes an “Achilles’ heel” in the structural genome of viral pathogens. Herein, we consider the potential of targeting such proteins to develop broad-spectrum therapeutics for the future. To illustrate this point, we outline the therapeutic potential of targeting the nonstructural protein 16 methyltransferase, which is conserved across most coronaviruses.

## 1. Introduction

**Figure 1:**
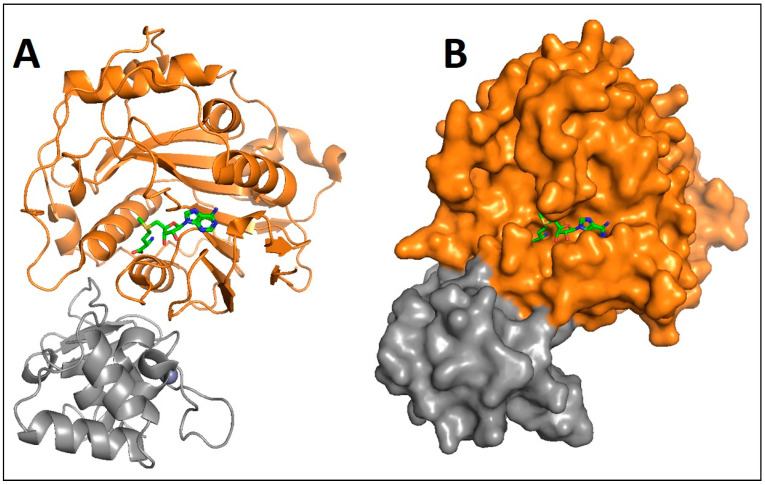
Ribbon (**A**) and Surface (**B**) representation of NSP16 (orange) in complex with NSP10 (grey). S-adenosylhomocysteine (green) is shown at the active site of NSP16. The crystal structure for the NSP16/10 complex was downloaded from the RCSB protein data bank (pdb ID 3R24) [5]. Figures were captured using MacPyMol.

The novel virus, severe acute respiratory syndrome coronavirus 2 (SARS-CoV-2) replicates in the host cytoplasm, underlying its dependence upon an autonomous mRNA capping mechanism (cap-1/m7GpppNm) [1]. This capping mechanism is achieved by the highly conserved nonstructural protein (NSP) 16, which is a 2′-O-methyltransferase (2′-O-MTase). The catalytic activity of this enzyme is essential both for replication in the host and for evasion of the host’s innate immune response, thereby allowing replication [1,2,3,4]. There is a high level of NSP16 structural conservation across most coronaviruses, including SARS-CoV (Figure 1) and Middle East respiratory syndrome coronavirus (MERS-CoV) [1,2,3,4]. Since all of these viruses have posed significant health risks in recent years, the structural conservation makes NSP16 an attractive therapeutic target for both the current pandemic as well as future outbreaks by yet-to-be discovered coronavirus variants.

## 2. Discussion and Conclusions

The Middle East respiratory syndrome coronavirus (MERS-CoV) NSP16 is an S-adenosyl-L-methionine (SAM)-dependent 2-O-methyltransferase (2-O-MTase) that methylates the ribose 2-OH of the first transcribed nucleotide (N1) of viral RNA cap structures. This 2-O-MTase activity is regulated by NSP10. The 2-O methylation prevents virus detection by innate cell immunity mechanisms and viral translation inhibition by the interferon-stimulated IFIT-1 protein. The mechanism for binding the RNA-substrate, SAM, capping the RNA substrate and releasing SAH is illustrated in Aouadi et al. 2017 [4]. The NSP16/NSP10 2-O-MTase activity is sensitive to known MTase inhibitors including sinefungin [2,4]. Thus, there is great potential in the identification of both active site and distal site inhibitors of NSP16 as a means of preventing coronaviruses from propagating.

The process of developing a broad-spectrum coronavirus methyltransferase inhibitor can be further facilitated by screening commercially available molecules from existing databases for virtual screening and molecular docking [6,7,8]. An example of this followed a similar approach targeting a 16N methyltransferase with molecular docking techniques through which raltegravir and maraviroc emerged as potential candidates for the treatment of coronavirus infections [9]. Both of these antiviral drugs were originally approved in 2007 for HIV indications. Thus, the repurposing of drugs combined with targeting-based drug development is an efficient methodology of drug development for future viral infections.

The greater value in targeting such an enzyme is that it is highly conserved across a range of viruses. As a result, in addition to developing a therapeutic that will target the coronavirus associated with the current outbreak, one would also be developing a broad-spectrum therapeutic that will be useful against known coronaviruses, future coronaviruses, and perhaps other groups of viral pathogens altogether. This “broad-spectrum” approach, when more broadly applied across a range of viral threats, represents a more efficient and comprehensive means of proactively managing future pandemics.
